# The impact of food additives, artificial sweeteners and domestic hygiene products on the human gut microbiome and its fibre fermentation capacity

**DOI:** 10.1007/s00394-019-02161-8

**Published:** 2019-12-18

**Authors:** Konstantinos Gerasimidis, Katie Bryden, Xiufen Chen, Eleftheria Papachristou, Anais Verney, Marine Roig, Richard Hansen, Ben Nichols, Rodanthi Papadopoulou, Alison Parrett

**Affiliations:** 1 Human Nutrition, School of Medicine, Dentistry and Nursing, College of Medical, Veterinary and Life Sciences, University of Glasgow, New Lister Building, Glasgow Royal Infirmary, Glasgow, G31 2ER UK; 2 Paediatric Gastroenterology, Hepatology and Nutrition, Royal Hospital for Children, 1345 Govan Road, Glasgow, G51 4TF UK

**Keywords:** Food additives, Microbiota, Fermentation capacity, Fibre, Gut microbiome

## Abstract

**Purpose:**

This study investigated the effect of food additives, artificial sweeteners and domestic hygiene products on the gut microbiome and fibre fermentation capacity.

**Methods:**

Faecal samples from 13 healthy volunteers were fermented in batch cultures with food additives (maltodextrin, carboxymethyl cellulose, polysorbate-80, carrageenan-kappa, cinnamaldehyde, sodium benzoate, sodium sulphite, titanium dioxide), sweeteners (aspartame-based sweetener, sucralose, stevia) and domestic hygiene products (toothpaste and dishwashing detergent). Short-chain fatty acid production was measured with gas chromatography. Microbiome composition was characterised with 16S rRNA sequencing and quantitative polymerase chain reaction (qPCR).

**Results:**

Acetic acid increased in the presence of maltodextrin and the aspartame-based sweetener and decreased with dishwashing detergent or sodium sulphite. Propionic acid increased with maltodextrin, aspartame-based sweetener, sodium sulphite and polysorbate-80 and butyrate decreased dramatically with cinnamaldehyde and dishwashing detergent. Branched-chain fatty acids decreased with maltodextrin, aspartame-based sweetener, cinnamaldehyde, sodium benzoate and dishwashing detergent. Microbiome Shannon α-diversity increased with stevia and decreased with dishwashing detergent and cinnamaldehyde. Sucralose, cinnamaldehyde, titanium dioxide, polysorbate-80 and dishwashing detergent shifted microbiome community structure; the effects were most profound with dishwashing detergent (*R*^2^ = 43.9%, *p* = 0.008) followed by cinnamaldehyde (*R*^2^ = 12.8%, *p* = 0.016). Addition of dishwashing detergent and cinnamaldehyde increased the abundance of operational taxonomic unit (OTUs) belonging to *Escherichia*/*Shigella* and *Klebsiella* and decreased members of Firmicutes, including OTUs of *Faecalibacterium* and *Subdoligranulum*. Addition of sucralose and carrageenan-kappa also increased the abundance of *Escherichia*/*Shigella* and sucralose, sodium sulphite and polysorbate-80 did likewise to *Bilophila*. Polysorbate-80 decreased the abundance of OTUs of *Faecalibacterium* and *Subdoligranulum*. Similar effects were observed with the concentration of major bacterial groups using qPCR. In addition, maltodextrin, aspartame-based sweetener and sodium benzoate promoted the growth of *Bifidobacterium* whereas sodium sulphite, carrageenan-kappa, polysorbate-80 and dishwashing detergent had an inhibitory effect.

**Conclusions:**

This study improves understanding of how additives might affect the gut microbiota composition and its fibre metabolic activity with many possible implications for human health.

**Electronic supplementary material:**

The online version of this article (10.1007/s00394-019-02161-8) contains supplementary material, which is available to authorized users.

## Introduction

A great amount of research has investigated the role of dietary nutrients, or dietary patterns in general, on the gut microbiome. Dietary fibre has attracted the most interest, mainly due to the inability of the human body to utilise it, and the capability of the gut microbiome to ferment it using a broad spectrum of enzymes not encoded in the human genome cannot encode [1]. Short-chain fatty acids (SCFA) are the end-product of fibre fermentation and the SCFA produced are dependent on the host’s diet and microbiome composition. Species within Bacteroides produce primarily acetic acid and propionic acid [2, 3]; members of *Clostridium leptum* cluster produce butyric acid from fibre fermentation and *Bifidobacterium* produces lactate and acetic acid from carbohydrate fermentation [4]. The branch chain fatty acids (BCFA) iso-butyric acid and iso-valeric acid are produced from protein breakdown, particularly in the absence of fermentable carbohydrate. Yet, the human gut microenvironment dynamics are more complex and characterised by an extensive degree of inter-species synergy and cross-feeding. It is, therefore, important to study the interactions between diet and the gut microbiome in the context of the entire microbial community and not as microbes in isolation. SCFA are critical bacterial products involved, not only locally in gut health, but in whole-body homeostasis. Along with an increased microbial diversity, high butyric acid concentration in the gut has been used as an indicator of healthy status of the microbiome. In contrast, reduced diversity, low luminal production of SCFA and dysbiosis have been proposed as primary events of inflammatory bowel disease, diabetes and obesity [5–8].

Our diet has evolved enormously and rapidly over the last century, in parallel with food preservation and processing and increased use of industrialised and domestic hygiene products. While food industrialisation has protected humanity from infectious diseases, the secondary effect this may have on gut microbiome-dependent host health, and the net impact on the incidence of non-communicable diseases has only relatively recently been considered. A Mediterranean diet with increased consumption of legumes, cereals, fruit and vegetables, and its health-promoting effects, influences the gut microbiome [9]. The Western diet, which includes food additives and preservatives, has contrastingly been associated with non-communicable diseases [10]. Food additives and artificial sweeteners have become increasingly prevalent within our diet, with more than 50% of available food in UK households being ultra-processed [11]. While food additives are evaluated rigorously for their effects on the host, health testing of food additives fails to include their effect on the human gut microbiome and by proxy long-term host health [12]. Recent studies in animals have indicated that food additives can have adverse effects on colonic and cardiovascular health, mediated by the gut microbiome and changes in the gut mucus layer. It has been shown that food emulsifiers, such as polysorbates and carboxymethyl cellulose can increase intestinal permeability, alter microbiota composition, promote *Escherichia coli* translocation across the epithelium and in M cells *in-vitro* causing gut inflammation [10, 13]. Likewise, the body of evidence on artificial sweeteners indicates that there are adverse metabolic outcomes in rodents owing to the onset of microbial dysbiosis [14–16]. Cumulative ingestion of residual products from regular use of domestic hygiene products may influence the human gut microbiome and, by extension, the health of the host. In epidemiological research, increased use of dishwashers, which reduce residual domestic detergent on dishware and consequent accidental ingestion, was associated with a decrease in cardiovascular disease [17]. Although for some food additives, artificial sweeteners and domestic hygiene products a large amount will be digested or degraded in the upper part of the gastrointestinal tract, residual amounts can still reach the colon. Others, like carrageenans and carboxylmethyl cellulose will reach the colon in similar amounts to those ingested.

It is, therefore, important to study the effect of food additives, artificial sweeteners and domestic hygiene products may have on gut microbiota composition and its fibre fermentation capacity, the most important bacterial function for host health. There is currently limited knowledge on the effect of additives on the human gut microbiota, and research to date has predominantly occurred in animal models with a paucity of evidence in humans. This preclinical study investigated the effect that commonly consumed food additives, including emulsifiers, artificial sweeteners and domestic hygiene products might have on the healthy human microbiota composition and its fibre fermentation capacity using *in-vitro* batch faecal fermentations.

## Subjects and methods

### Participants

Thirteen young healthy adults (females, *n* = 7; mean, (SD); age: 24.8, (2.2) years; body mass index (BMI) 21.9, (2.8) kg/m^2^) donated a single faecal sample. Participants who had used antibiotics within the three months prior were not eligible to participate. Participants provided informed consent. The study received ethical approval by the Medical, Veterinary and Life Sciences Research Ethics Committee, at the University of Glasgow.

### In-vitro batch faecal fermentation studies

Faecal samples were collected in disposable containers and processed within one hour of defecation. From each donor, a faecal slurry (16% w/v) was prepared using 16 g of faecal matter homogenised in 100 ml Sorensen’s buffer pH 7, boiled and degassed under oxygen-free nitrogen stream. The faecal slurry was strained through 30-denier nylon stockings to remove coarse material and remained in suspension by continuous agitation using a magnetic stirrer. In a 150 ml flask, 5 ml of 16% faecal slurry were added along with 42 ml of in-house prepared fermentation medium, 2 ml of reducing solution, 400 mg of fibre substrate (see below) and one of the additives in testing. Assuming that an average person has a faecal output of 120 g/day [18] and a recommended intake of the fibre of 30 g/day, this would be equivalent to roughly double the amount of fibre available for fermentation per g of faeces.

The fermentation medium was prepared in-house (1 litre). It consisted of 225 ml of macromineral solution (0.04 M Na_2_HPO_4_, 0.046 M KH_2_PO_4_, 0.002 M MgSO_4_·7H_2_O), 225 ml buffer solution (0.051 M NH_4_HCO_3_ and 0.417 M NaHCO_3_), 112.5 μl of micromineral solution (0.898 M CaCl_2_·2H_2_O, 0.505 M MnCl_2_·4H_2_O, 0.042 M CoCl_2_·6H_2_O, and 0.296 M FeCl_3_·6H_2_O), 1.125 ml of 0.1% resazurin solution, 450 ml of 5 mg/mL Tryptone, 100 mg of mucin from porcine stomach, and 76 mg of mixed bile extract from porcine. Once the solution was made, it was boiled, degassed under oxygen-free nitrogen, and adjusted to pH 7 to mimic the distal intestinal environment. Reducing solution (50 ml) was made up of 2 ml of 1 M NaOH, 312.5 mg of cysteine hydrochloride and 312.5 mg of Na_2_S·9H_2_O.

The fibre substrate was made up of 100 mg of apple pectin (SIGMA, Pectin, from apple), 100 mg of raftilose (Beneo™, Orafti P95), 100 mg of α-cellulose (SIGMA™, α-CELLULOSE), and 100 mg of high resistant maize starch (National StarchTM, HI-MAIZE[TM] 260). We chose these fibres as indicative of food consumed in the UK diet [19].

Eight food additives [maltodextrin, carboxymethyl cellulose, polysorbate-80, carrageenan-kappa, sodium benzoate, sodium sulphite, titanium dioxide, cinnamaldehyde], three artificial sweeteners [aspartame-based sweetener, sucralose, stevia], and two domestic hygiene products [toothpaste, dishwashing detergent] were used. Test amounts were based on the acceptable daily intake or estimated daily consumption, assuming an average male adult weighing 75 kg (Online Resource 1). Where the estimated daily consumption was relatively large (maltodextrin, carboxymethyl cellulose, polysorbate-80, carrageenan-kappa, aspartame-based sweetener), the amount tested was standardised to 500 mg. Likewise, where estimated daily consumption was relatively small (stevia, cinnamaldehyde, sodium benzoate, sodium sulphite, sucralose), the amount tested was 50% of the acceptable daily intake. For the toothpaste and the dishwashing detergent, the amount tested was 100% of estimated accidental intake (Online Resource 1). Selection of additives was based on previous research which implicated them in the onset of non-communicable diseases including inflammatory bowel disease and metabolic syndrome [10, 20, 21].

Thirteen fermentation flasks, one for each of the additives above, and a non-additive blank (hereafter referred to as control) were degassed under oxygen-free nitrogen stream and incubated in a shaking water bath at 37 °C at 60 strokes/min for 24 h. A baseline sample was collected from the control prior to incubation start and from all other additives and the control after 24 h of incubation. Aliquots of fermentation slurry for SCFA analysis were collected and stored in 3:1 ratio with 1 M NaOH at − 20 °C until analysis. Fermentation slurry aliquots were stored at -80 °C and total DNA was extracted within a month of collection.

### Measurement of net SCFA production

The SCFA (acetic acid, propionic acid, butyric acid, valeric acid, caproic acid, heptanoic acid, and caprylic acid) and BCFA (iso-butyric acid and iso-valeric acid) were extracted from acidified slurries three times in total using diethyl ether. Extracts were analysed using Gas Chromatography (Agilent 7890A) with flame ionisation detector, as described previously [22, 23]. Each of the SCFA was quantified against calibration curves plotted using authentic external standards [acetic acid (185.8 mM), propionic acid (144.5 mM), butyric acid (114.2 mM), valeric acid (83.4 mM), caproic acid (52.6 mM), heptanoic acid (65.8 mM), caprylic acid (53.2 mM), isobutyric acid (97.3 mM), and isovaleric acid (87.0 mM) all stored in 2 M NaOH and using 2-ethylbutyric acid (74.0 mM) as internal standard. All samples from the same participant were analysed in the same run to minimise inter-assay variation. Each sample was measured twice, and in all cases the average concentration was calculated unless the % co-efficient of variation was greater than 10% in which case a third replicate was analysed. Concentration of SCFA (μmol) is reported per volume (ml) of fermentation slurry.

### Extraction of genomic DNA from fermentation slurries

In a subset of 8 participants, 16S rRNA amplicon sequencing of the human gut microbiome and quantification of total and 5 dominant bacterial groups were performed. Samples were thawed at room temperature and after centrifugation at 12,000 *g* for 5 min, genomic DNA from the resultant pellet was extracted using the DNeasy Powersoil Kit. The purity and concentration of extracted DNA was quantified using the NanoDrop™ 1000 and Qubit.

### Quantification of dominant bacterial groups of the human gut microbiome

Quantitative PCR (qPCR) was performed using TaqMan™ chemistry and quantified against serial dilution of standards prepared from pure bacterial cultures as described previously [22]. Total bacteria and 5 different bacterial groups were targeted (Bacteroides/Prevotella, *Bifidobacterium*, *Blautia coccoides*, *Clostridium leptum* and *E. coli*) (Online Resource 2). The PCR reaction consisted of 7.5 µl Taqman™ gene expression master mix, 2.25 µl nuclease-free water, 0.5 µl bovine serum albumin, 1.5 µl forward primer (9 µM), 1.5 µl reverse primer (9 µM) and 0.75 µl probe (2.5 µM). qPCR was performed in triplicates and averages calculated for replicates where Ct difference was less than 0.2 Ct.

### Characterisation of global microbiome with 16S rRNA sequencing

Sequencing of the V4 region of the 16S rRNA gene was performed on the MiSeq (Illumina, Essex, UK) platform using 2 × 250 bp paired-end reads [23, 24].

### Bioinformatics

To enable analysis of the gut microbiome, 97% operational taxonomic units (OTUs) were generated from the 16S rRNA sequences using an adaptation of the VSEARCH pipeline (https://github.com/torognes/vsearch/wiki/VSEARCH-pipeline) [25]. Quality filtering was performed on the combined paired reads with a maximum allowed expected error rate of 0.5 base pairs per read. Sequences longer than 275 bp and shorter than 225 bp were also filtered out. The next steps involved dereplication, removal of singleton sequences and preclustering at 98%. Chimeras were removed using the VSEARCH implementation of the UCHIME *de-novo* algorithm followed by the UCHIME reference-based method in conjunction with the 'Gold' ChimeraSlayer reference dataset [26, 27]. Finally, OTUs were assigned by clustering the remaining sequences at 97% and taxonomically classified using a naive Bayesian classifier method implemented in the dada2 R package [28].

### Statistical analysis

Data are presented as medians and interquartile (Q1–Q3) range. One-sample Wilcoxon (non-normally distributed data) or paired *t *test (normally distributed data) was used to calculate the difference between each additive and the control. Microbiome analysis using the 16S rRNA gene sequences was carried out in R version 3.5.3. The alpha diversity measures (i.e. rarefied richness, Chao1 richness estimate, Shannon diversity index, and Pielou's evenness) were all calculated using the vegan package [29]. Permutation ANOVA results were also generated using vegan on both Bray–Curtis and UniFrac distance matrices. In the case of UniFrac the phylogenetic tree was generated using FastTree 2 [30]. Nonmetric multidimensional scaling (NMDS) was performed with the phyloseq package [31] and was used to visualise overall community structure in the form of ordination plots. Differentially abundant taxa were found using paired t-tests on log-relative abundances. Only significant differences greater than 0.5 logs are reported. Significance was set at 0.05.

## Results

### Effect of additives on net SCFA production

**Fig. 1:**
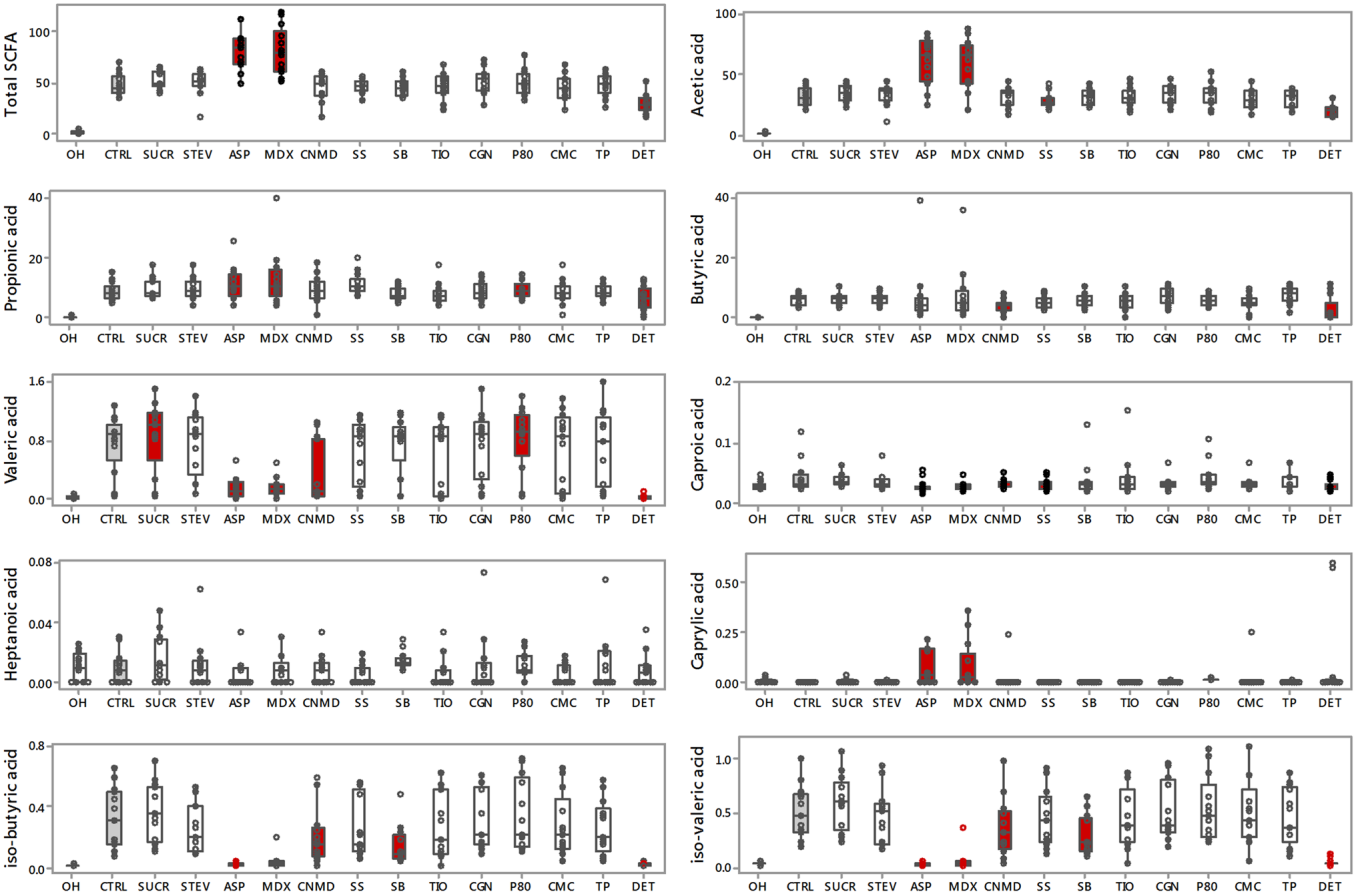
Baseline and net production of total and individual short chain fatty acids (μmol/ml) following 24 h batch faecal fermentation of fibre with food additives, artificial sweeteners and domestic hygiene products. Red filling boxplot indicates significant difference (*p* < 0.05) compared with the CTRL (displayed with grey filling boxplot); *0H* baseline, *CTRL* control, *SUCR* sucralose, *STEV* stevia, *ASP* aspartame based sweetener, *MDX* maltodextrin, *CNMD* cinnamaldehyde, *SS* sodium sulphite, *SB* sodium benzoate, *TIO* titanium dioxide, *CGN* carrageenan-kappa, *P80* polysorbate-80, *CMC* carboxymethyl cellulose, *TP* toothpaste, *DET* detergent

Figure 1 displays the net production of SCFA and Table 1 the median of the difference in their concentration with respect to the 24 h control, for each additive. Fermentation of the control for 24 h increased the production of total SCFA (0 h vs 24 h; 1.73 vs 45.36, µmol/ml; *p* < 0.0001) (Fig. 1). Addition of maltodextrin and aspartame-based sweetener produced the highest median concentration of total SCFA whereas the dishwashing detergent the lowest (Fig. 1). Considering the individual SCFA, maltodextrin (*p* < 0.001) and aspartame-based sweetener (*p* < 0.001) increased the production of acetic acid whilst in contrast, dishwashing detergent (*p* < 0.001) and sodium sulphite (*p* = 0.036) caused a significant decrease in acetic acid production compared with the control (Fig. 1). Production of propionic acid was increased when maltodextrin (*p* = 0.014), polysorbate-80 (*p* = 0.044), sodium sulphite (*p* = 0.011) or the aspartame-based sweetener (*p* = 0.034) were present (Fig. 1). Addition of cinnamaldehyde (*p* = 0.006) or dishwashing detergent (*p* = 0.012) significantly decreased the production of butyric acid when compared with the control; a similar non-significant effect (*p* = 0.052) was also observed for sodium sulphite (Fig. 1). Compared with the control, sucralose (*p* = 0.025) and polysorbate-80 (*p* = 0.003) significantly increased production of valeric acid whereas when maltodextrin (*p* = 0.002), cinnamaldehyde (0.014), aspartame-based sweetener (*p* = 0.002) and the dishwashing detergent (*p* = 0.002) were added a significant decrease was observed (Fig. 1). There was a significant decrease in the production of caproic acid when maltodextrin (*p* = 0.012), cinnamaldehyde (*p* = 0.021), sodium sulphite (*p* = 0.014), aspartame-based sweetener (*p* = 0.002) or dishwashing detergent (*p* = 0.010) were added (Fig. 1). Caprylic acid significantly increased in the presence of maltodextrin (*p* = 0.006), polysorbate-80 (*p* = 0.002), aspartame-based sweetener (*p* = 0.009) and dishwashing detergent (0.035) (Fig. 1). With regard to the BCFA, there was a significant decrease in the production of isobutyric acid when maltodextrin (*p* = 0.002), cinnamaldehyde (*p* = 0.025), sodium butyrate (*p* = 0.014), aspartame-based sweetener (*p* < 0.001) or dishwashing detergent (*p* = 0.002) were added (Fig. 1). Similar effects were also seen for isovaleric acid [maltodextrin (*p* = 0.002), cinnamaldehyde (*p* = 0.041), sodium benzoate (*p* = 0.004), aspartame-based sweetener (*p* < 0.001) and dishwashing detergent (*p* = 0.002)] (Fig. 1). Carboxymethyl cellulose, toothpaste, carrageenan-kappa, titanium dioxide, sodium benzoate and stevia had no effect on the production of any SCFA or BCFA. The effect of each of the substrates on the proportional ratio or SCFA are displayed in Online Resource 3.

**Table 1:** Difference (from non-additive control), in the net production of total and individual short chain fatty acids (μmol/ml) following 24 h batch faecal fermentation of fibre with food additives, artificial sweeteners and domestic hygiene products

SCFA	SUCR	MDX	STEV	CNMD	CMC	P80	CGN	SB	SS	TIO	ASP	TP	DET
*Total SCFA*													
Median	3.870	28.460	2.450	− 3.560	− 2.460	1.390	2.010	− 3.410	− 2.240	0.710	30.820	− 0.700	− 17.010
Q1	− 0.750	17.750	− 3.620	− 8.880	− 13.780	− 2.300	− 0.720	− 6.860	− 5.750	− 7.730	25.730	− 1.940	− 19.810
Q3	6.140	45.070	8.940	7.840	9.850	5.400	6.650	2.100	2.370	3.160	40.970	2.950	− 13.570
*p* value	0.107*	< 0.001*	0.402	0.539*	0.492*	0.232*	0.100*	0.289*	0.374*	0.571*	< 0.001*	0.922*	< 0.001*
*Acetic acid*													
Median	2.300	29.810	0.320	− 0.360	− 1.000	1.350	0.730	− 1.160	− 3.440	0.130	31.550	− 1.537	− 10.110
Q1	− 0.620	16.800	− 3.990	− 5.560	− 8.890	− 1.220	0.040	− 4.070	− 5.920	− 1.880	23.100	− 2.500	− 12.570
Q3	4.720	44.110	6.530	4.850	6.050	3.310	4.540	3.720	− 0.280	2.380	40.040	1.324	− 7.460
*p* value	0.081*	< 0.001*	0.625	0.919*	0.581*	0.219*	0.070*	0.475*	0.036*	0.783*	< 0.001*	0.104*	< 0.001*
*Propionic acid*													
Median	0.472	1.900	0.776	1.447	− 0.427	0.257	− 0.349	− 0.873	1.760	− 0.641	1.860	0.000	− 2.610
Q1	− 0.621	0.350	− 0.052	− 0.956	− 3.142	− 0.178	− 0.997	− 1.989	1.234	− 3.019	0.940	− 0.497	− 5.400
Q3	1.116	4.380	1.380	2.870	1.241	1.057	1.639	0.401	5.910	0.397	3.500	0.678	0.900
*p* value	0.364	**0.014**	0.277*	0.437*	0.665*	0.044*	0.889*	0.218*	0.011*	0.235	0.034*	0.849*	0.071*
*Butyric acid*													
Median	0.647	− 1.270	1.055	− 1.959	− 1.064	− 0.637	0.958	0.138	− 0.735	0.014	− 1.240	1.237	− 4.770
Q1	− 0.325	− 3.940	− 0.869	− 3.462	− 2.608	− 0.965	0.228	− 2.055	− 2.754	− 2.196	− 3.390	0.221	− 6.540
Q3	1.197	4.740	1.285	− 0.426	0.943	0.279	1.670	1.277	− 0.092	0.447	1.590	2.887	− 0.320
*p* value	0.570*	1.000	0.445*	0.006*	0.252*	0.268*	0.125*	0.763*	0.052*	0.230*	0.328	0.102*	**0.012**
*Valeric acid*													
Median	0.045	− 0.655	0.011	− 0.120	0.020	0.063	0.010	− 0.018	− 0.005	0.005	− 0.669	0.067	− 0.837
Q1	0.005	− 0.785	− 0.163	− 0.050	− 0.060	0.014	− 0.050	− 0.053	− 0.126	− 0.285	− 0.786	− 0.097	− 0.956
Q3	0.186	− 0.400	0.080	0.070	0.108	0.133	0.070	0.054	0.130	0.052	− 0.445	0.115	− 0.513
*p* value	**0.025**	**0.002**	0.834	**0.014**	0.834	**0.003**	0.485	0.727	0.675	0.675	**0.002**	0.529	**0.002**
*Caproic acid*													
Median	0.001	− 0.006	0.000	− 0.002	− 0.001	0.002	− 0.001	− 0.003	− 0.005	− 0.003	− 0.007	0.001	− 0.006
Q1	− 0.004	− 0.021	− 0.004	− 0.012	− 0.013	− 0.003	− 0.007	− 0.009	− 0.016	− 0.008	− 0.019	− 0.009	− 0.021
Q3	0.006	− 0.001	0.002	0.000	0.001	0.006	0.003	0.001	0.001	0.003	− 0.005	0.003	− 0.001
*p* value	0.576	**0.012**	0.780	**0.021**	0.162	0.402	0.485	0.234	**0.014**	0.328	**0.002**	0.727	**0.010**
*Heptanoic acid*													
Median	0.004	0.000	0.001	0.000	0.000	0.004	0.000	0.009	− 0.003	0.000	− 0.005	0.000	− 0.004
Q1	− 0.005	− 0.007	− 0.005	− 0.013	− 0.011	− 0.001	− 0.007	0.001	0.009	− 0.006	− 0.012	− 0.006	− 0.009
Q3	0.018	0.005	0.008	0.010	0.002	0.009	0.002	0.012	0.000	0.002	0.000	0.006	0.008
*p* value	0.307	0.813	0.625	0.756	0.476	0.465	0.906	0.142	0.107	0.407	0.221	1.000	0.689
*Caprylic acid*													
Median	0.000	0.022	0.000	0.000	0.000	0.011	0.000	0.000	0.000	0.000	0.033	0.000	0.000
Q1	0.000	0.002	0.000	0.000	0.000	0.007	0.000	0.000	0.000	0.000	0.000	0.000	0.000
Q3	0.009	0.147	0.002	0.000	0.000	0.014	0.000	0.000	0.000	0.000	0.165	0.000	0.015
*p* value	0.142	**0.006**	0.201	0.584	1.000	**0.002**	0.361	1.000	0.423	0.371	**0.009**	0.584	**0.035**
*Iso butyric acid*													
Median	0.013	− 0.293	− 0.046	− 0.076	− 0.001	0.011	0.013	− 0.104	− 0.054	− 0.005	− 0.296	− 0.012	− 0.274
Q1	− 0.057	− 0.468	− 0.170	− 0.258	− 0.089	− 0.061	− 0.078	− 0.343	− 0.174	− 0.107	− 0.472	− 0.223	− 0.487
Q3	0.046	− 0.100	0.029	0.017	0.050	0.093	0.051	− 0.063	0.035	0.011	− 0.126	0.046	− 0.113
*p* value	0.375*	**0.002**	0.163*	**0.025**	0.944	0.780	0.870*	0.014*	0.184	0.211*	< 0.001*	0.343*	**0.002**
*Isovaleric acid*													
Median	0.002	− 0.461	− 0.078	− 0.126	0.047	0.019	− 0.005	− 0.220	− 0.177	− 0.019	− 0.463	− 0.037	− 0.425
Q1	− 0.038	− 0.649	− 0.125	− 0.269	− 0.015	− 0.107	− 0.119	− 0.376	− 0.018	− 0.113	− 0.645	− 0.196	− 0.642
Q3	0.124	− 0.253	0.044	0.039	0.105	0.167	0.075	− 0.115	0.072	0.032	− 0.301	0.093	− 0.260
*p* value	0.208*	**0.002**	0.487*	0.041*	0.895*	0.759*	0.675	0.004*	0.266*	0.338*	< 0.001*	0.614*	**0.002**

Data are presented as in µmol/ml of faecal slurry; 1-sample Wilcoxon test was used for non-parametric data and a paired *t-*test for parametric data (indicated with asterisk); with bold fonts are displayed statistically significant differences (*p* < 0.05)

*SUCR* sucralose, *MDX* maltodextrin, *STEV* stevia, *CNMD* cinnamaldehyde, *CMC* carboxymethyl cellulose, *P80* polysorbate 80, *CGN* carrageenan, *SB* sodium benzoate, *SS* sodium sulphite, *TIO* titanium dioxide, *ASP* aspartame- based sweetener, *TP* toothpaste, *DET* detergent

### Effect of additives on microbiome diversity indices

**Fig. 2:**
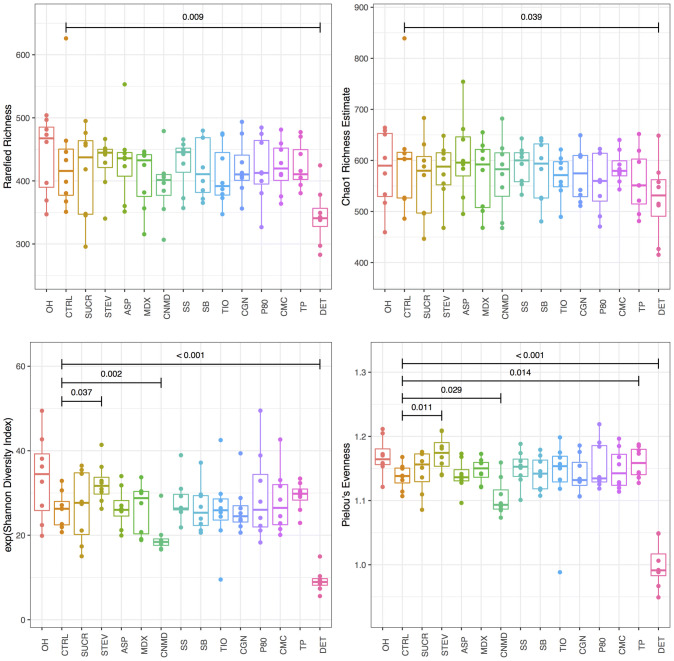
Microbiome α-diversity indices before and following 24 h batch faecal fermentation of fibre with food additives, artificial sweeteners and domestic hygiene products. *0H* baseline, *CTRL* control, *SUCR* sucralose, *STEV* stevia, *ASP* aspartame based sweetener, *MDX* maltodextrin, *CNMD* cinnamaldehyde, *SS* sodium sulphite, *SB* sodium benzoate, *TIO* titanium dioxide, *CGN* carrageenan-kappa, *P80* polysorbate-80, *CMC* carboxymethyl cellulose, *TP* toothpaste, *DET* detergent

Compared to the control group, the addition of dishwashing detergent significantly decreased all metrics of microbiome α-diversity, including OTU richness, evenness and the Shannon diversity index (Fig. 2). Incubation of faecal microbiota with cinnamaldehyde decreased the Shannon diversity index whereas an effect in the opposite direction was provoked by stevia. The effects of stevia and cinnamaldehyde on Shannon diversity index were due to an effect on microbiome community evenness rather than an impact on OTU richness (Fig. 2). There were no other significant effects on α-diversity indices for the rest of the substrates.

### Effect of additives on microbiome community structure

**Fig. 3:**
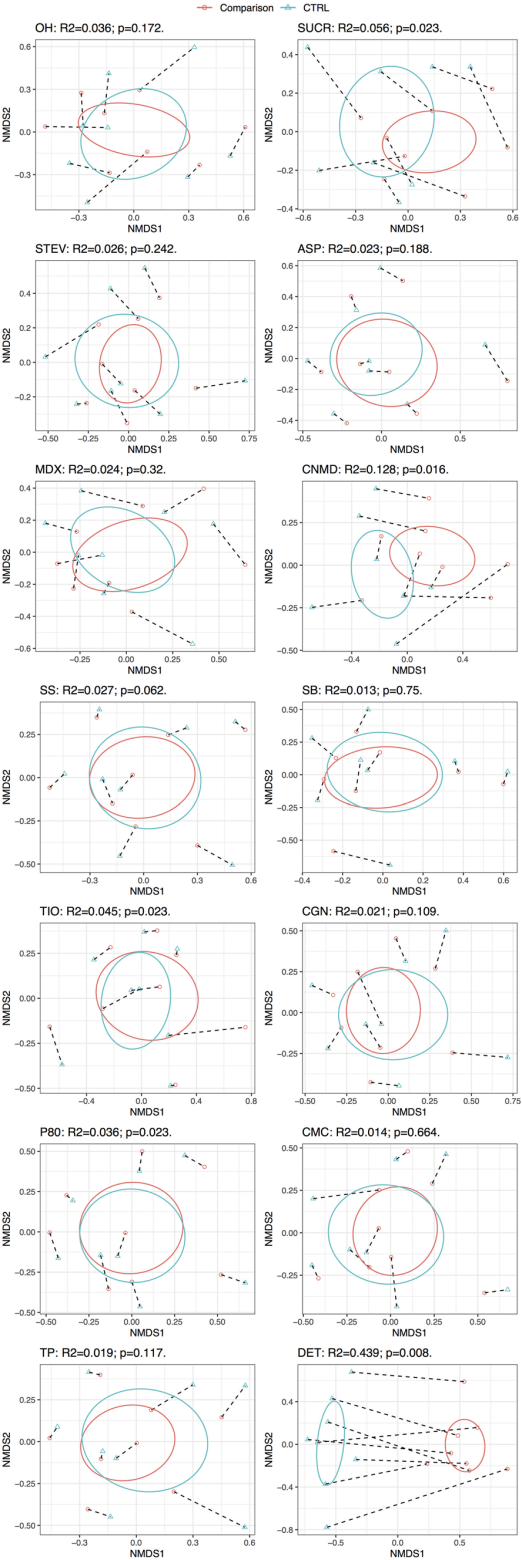
Microbiome community structure (β diversity) using the Bray–Curtis dissimilarity index before and following 24 h batch faecal fermentation of fibre with food additives, artificial sweeteners and domestic hygiene products. *0H* baseline, *CTRL* control, *SUCR* sucralose, *STEV* stevia, *ASP* aspartame based sweetener, *MDX* maltodextrin, *CNMD* cinnamaldehyde, *SS* sodium sulphite, *SB* sodium benzoate, *TIO* titanium dioxide, *CGN* carrageenan-kappa, *P80* polysorbate-80, *CMC* carboxymethyl cellulose, *TP* toothpaste, *DET* detergent

**Fig. 4:**
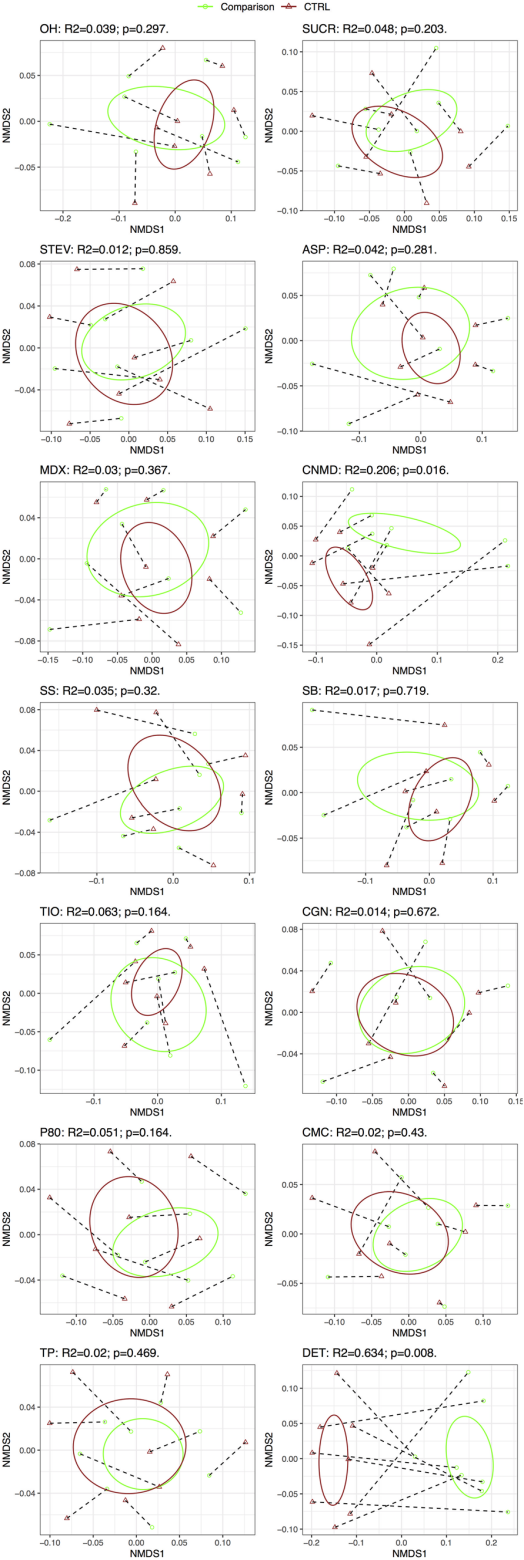
Microbiome community structure (β diversity) using the UniFrac unweighted distances before and following 24 h batch faecal fermentation of fibre with food additives, artificial sweeteners and domestic hygiene products. *0H* baseline, *CTRL* control, *SUCR* sucralose, *STEV* stevia, *ASP* aspartame based sweetener, *MDX* maltodextrin, *CNMD* cinnamaldehyde, *SS* sodium sulphite, *SB* sodium benzoate, *TIO* titanium dioxide, *CGN* carrageenan-kappa, *P80* polysorbate-80, *CMC* carboxymethyl cellulose, *TP* toothpaste, *DET* detergent

Addition of sucralose, cinnamaldehyde, titanium dioxide, polysorbate-80 and dishwashing detergent induced significant shifts in microbiome community structure (β-diversity) using the Bray–Curtis dissimilarity index (Fig. 3). The most pronounced effects were from dishwashing detergent followed by cinnamaldehyde, which explained 43.9% (*p* = 0.008) and 12.8% (*p* = 0.016) of the variance in microbiome community structure, respectively. The effects of sucralose (*R*^2^ = 5.6%, *p* = 0.023), polysorbate-80 (*R*^2^ = 3.6%, *p* = 0.023) and titanium dioxide (*R*^2^ = 4.5%, *p* = 0.023) were significant but less pronounced. When we looked at the effects of food additives, artificial sweeteners and domestic hygiene products on their microbiome community structure using UniFrac distances, which consider OTU phylogenetic relatedness, a significant effect was observed for cinnamaldehyde (*R*^2^ = 20.6%, *p* = 0.016) and dishwashing detergent (*R*^2^ = 63.4%, *p* = 0.008) (Fig. 4). The effect of dishwashing liquid and cinnamaldehyde on microbiome community structure dominated that of inter-subject variation (Figs. 3, 4).

### Effect of additives on taxon relative abundance

**Fig. 5:**
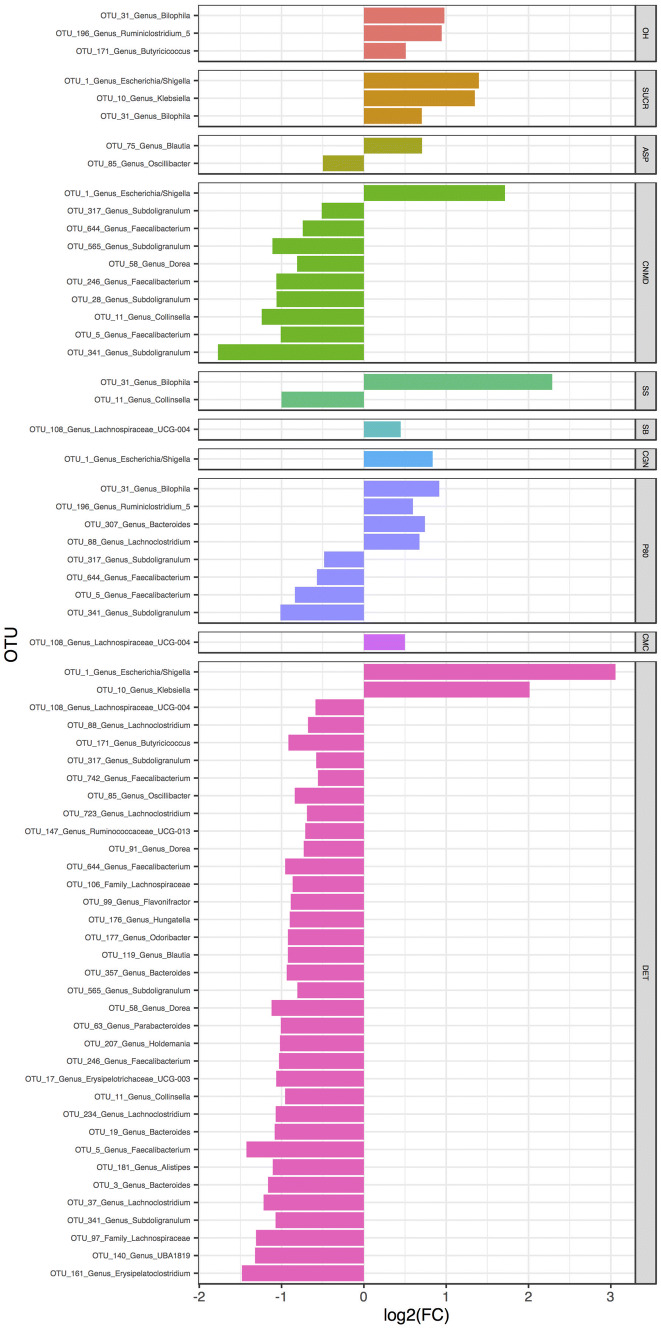
The effect of food additives, artificial sweeteners and domestic hygiene products on bacterial OTU relative abundance. *0H* baseline, *CTRL* control, *SUCR* sucralose, *STEV* stevia, *ASP* aspartame based sweetener, *MDX* maltodextrin, *CNMD* cinnamaldehyde, *SS* sodium sulphite, *SB* sodium benzoate, *TIO* titanium dioxide, *CGN* carrageenan-kappa, *P80* polysorbate-80, *CMC* carboxymethyl cellulose, *TP* toothpaste, *DET* detergent, *log2(FC)* log2 fold change

In accordance with the significant shifts observed on α and β diversity, major effects in taxon relative abundance were observed with the fermentation of fibre in the presence of cinnamaldehyde and dishwashing detergent (Fig. 5). Addition of dishwashing detergent increased the relative abundance of OTU belonging to *Escherichia*/*Shigella* and *Klebsiella* and in parallel decreased the relative abundance of 33 other OTUs, the majority of which belonged to Firmicutes. A similar increase of an OTU of *Escherichia*/*Shigella* was observed for cinnamaldehyde whereas 9 other OTUs, including three of *Faecalibacterium* and four of *Subdoligranulum*, all important butyrate producers, significantly decreased (Fig. 5). The relative abundance of *Escherichia*/*Shigella* also increased in the presence of sucralose and carrageenan-kappa. Similarly, a species of *Bilophila* increased with the addition of sucralose, sodium sulphite and polysorbate-80. Except for dishwashing detergent and cinnamaldehyde, major declines in the abundance of OTUs of *Faecalibacterium* and *Subdoligranulum* were observed using polysorbate-80 as substrate. There was no effect on the addition of maltodextrin, stevia, titanium dioxide and toothpaste on OTU relative abundance (Fig. 5). Similar effects were observed at genus and at the family level (Online Resource 4).

### Effect of additives on the growth of major bacterial groups

**Fig. 6:**
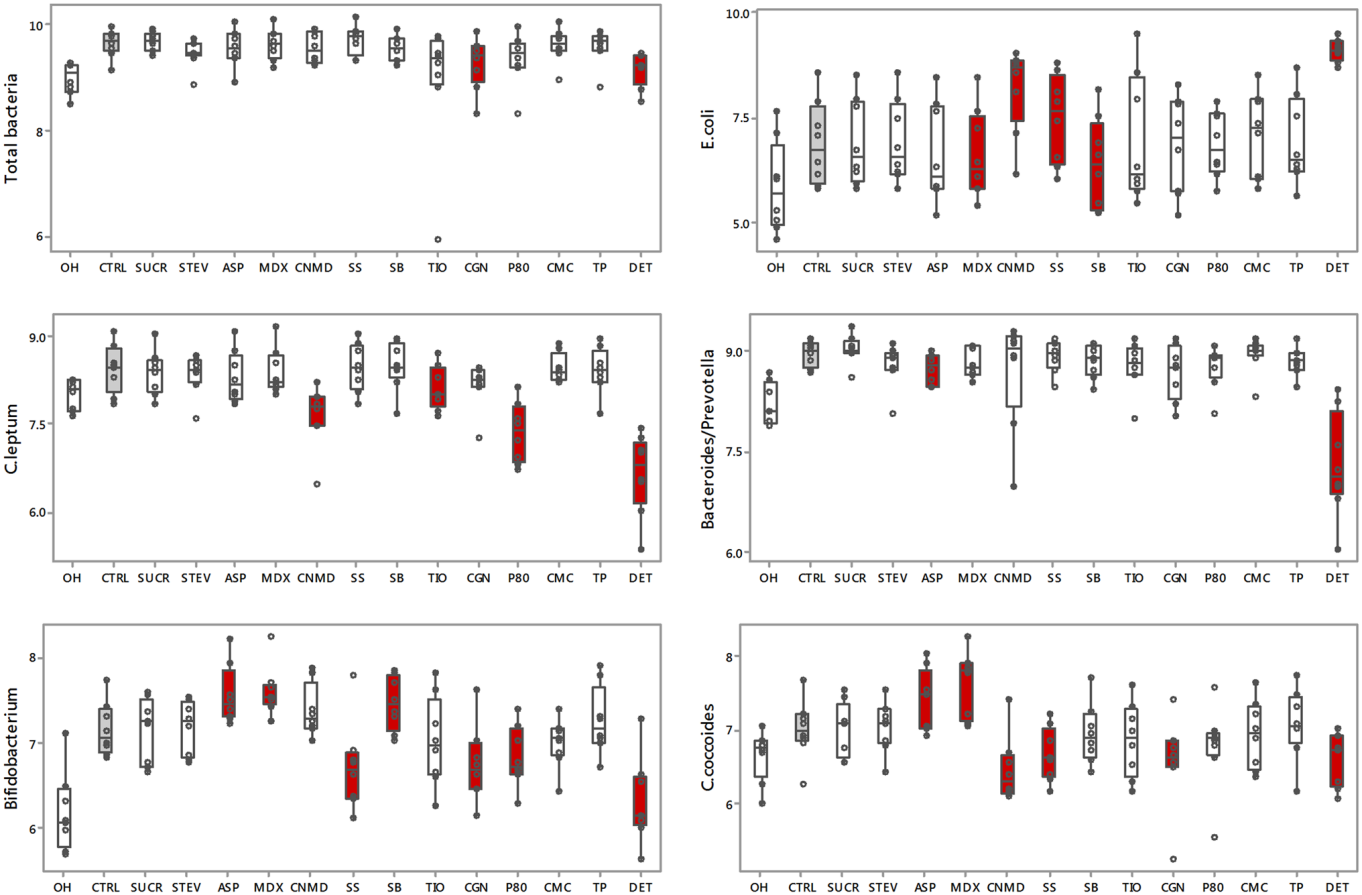
Concentration of total and major bacterial groups (number of 16S rRNA gene copies/ml) before and following 24 h batch faecal fermentation of fibre with food additives, artificial sweeteners and domestic hygiene products. Red filling boxplot indicates significant difference (*p* < 0.05) compared with the CTRL (displayed with grey filling boxplot);* 0H* baseline, *CTRL* control, *SUCR* sucralose, *STEV* stevia, *ASP* aspartame based sweetener, *MDX* maltodextrin, *CNMD* cinnamaldehyde, *SS* sodium sulphite, *SB* sodium benzoate, *TIO* titanium dioxide, *CGN* carrageenan-kappa, *P80* polysorbate-80, *CMC* carboxymethyl cellulose, *TP* toothpaste, *DET* detergent

Figure 6 shows the absolute concentration and Table 2 the median difference of 16S rRNA gene copy number, between the various additives and the control, for each bacterial group tested. Regardless of the type of additive tested, the concentration of total bacteria significantly increased after 24 h fermentation and Bacteroides/Prevotella and *C. leptum* cluster typically represented the two most dominant groups (Fig. 6). Among the additives, the addition of carrageenan-kappa (*p* = 0.034) and dishwashing detergent (*p* = 0.002), significantly decreased the concentration of total bacteria in comparison with the control group (Fig. 6). Similarly, maltodextrin (*p* = 0.021) and sodium benzoate (*p* < 0.001) significantly decreased the concentration of *E. coli* whereas addition of cinnamaldehyde (*p* = 0.014), sodium sulphite (*p* = 0.038) or dishwashing detergent (*p* < 0.001) promoted their growth (Fig. 6). The growth of species belonging to *C. leptum* significantly decreased in the presence of cinnamaldehyde (*p* = 0.003), polysorbate-80 (*p* = 0.001), titanium dioxide (*p* = 0.029) and dishwashing detergent (*p* < 0.001) and it was also the case for Bacteroides/Prevotella, when aspartame-based sweetener (*p* = 0.048) or dishwashing detergent were added (*p* = 0.001) (Fig. 6). *Bifidobacterium* growth increased from the control with the addition of maltodextrin (*p* = 0.002), sodium benzoate (*p* = 0.008) and aspartame-based sweetener (*p* = 0.005) (Fig. 6). In contrast, a significant inhibitory effect on *Bifidobacterium* was observed with polysorbate-80 (*p* = 0.036), carrageenan-kappa (*p* = 0.003), sodium sulphite (*p* = 0.013) or dishwashing detergent (*p* < 0.001) (Fig. 6). When compared with the control group, cinnamaldehyde (*p* = 0.003), carrageenan-kappa (*p* = 0.014), sodium sulphite (*p* = 0.001) and dishwashing detergent (*p* = 0.014) significantly inhibited the growth of the *B. coccoides* group whereas maltodextrin (*p* = 0.002) and aspartame-based sweetener (*p* = 0.009) significantly promoted this (Fig. 6). Stevia, sucralose, carboxymethyl cellulose and toothpaste had no significant effects on the growth of these broad bacterial populations (Fig. 6).

**Table 2:** Difference (from control) in the concentration of total and major bacterial groups (number of 16S rRNA gene copies/ml) following 24 h batch faecal fermentation of fibre with food additives, artificial sweeteners and domestic hygiene products

Bacterial group	SUCR	MDX	STEV	CNMD	CMC	P80	CGN	SB	SS	TIO	ASP	TP	DET
*Total bacteria*													
Median	0.005	0.034	− 0.153	− 0.091	0.020	− 0.271	− 0.320	− 0.086	0.044	− 0.212	− 0.123	− 0.060	− 0.432
Q1	− 0.082	− 0.263	− 0.236	− 0.332	− 0.186	− 0.508	− 0.728	− 0.303	− 0.148	− 0.863	− 0.356	− 0.225	− 0.739
Q3	0.194	0.222	− 0.064	0.184	0.220	0.084	− 0.227	0.140	0.160	− 0.074	0.173	0.026	− 0.256
*p* value	0.393*	0.877*	0.140*	0.387*	0.755*	0.114*	0.034*	0.295*	0.485*	0.141*	0.377*	0.294	0.002*
*E. coli*													
Median	− 0.042	− 0.165	− 0.003	1.224	0.030	0.000	− 0.223	− 0.404	0.406	− 0.001	− 0.310	0.116	2.298
Q1	− 0.195	− 0.547	− 0.036	0.393	− 0.059	− 0.086	− 0.372	− 0.550	0.218	− 0.561	− 0.624	− 0.126	1.269
Q3	0.305	− 0.054	0.144	2.235	0.563	0.175	0.035	− 0.360	0.597	0.077	− 0.031	0.238	3.208
*p* value	0.816*	0.021*	0.667*	**0.014**	0.149*	0.856*	0.861*	< 0.001*	0.038*	0.853*	0.101*	0.689*	< 0.001*
*C. leptum*													
Median	− 0.185	− 0.046	− 0.022	− 0.915	− 0.070	− 1.014	− 1.014	0.082	0.051	− 0.214	− 0.083	− 0.016	− 1.746
Q1	− 0.360	− 0.290	− 0.603	− 1.132	− 0.180	− 1.600	− 0.604	− 0.151	− 0.038	− 0.535	− 0.429	− 0.196	− 2.343
Q3	0.397	0.222	0.281	− 0.551	0.247	− 0.717	− 0.066	0.407	0.202	− 0.074	0.119	0.058	− 1.393
*p* value	0.642*	0.615*	0.834	0.003*	0.898*	0.001*	0.800	0.809*	0.770*	0.029*	0.191*	0.683*	< 0.001*
*Bacteroides/Prevotella*													
Median	0.083	− 0.067	− 0.104	0.066	0.013	− 0.148	− 0.266	− 0.072	0.034	− 0.041	− 0.219	− 0.129	− 1.758
Q1	− 0.027	− 0.312	− 0.291	− 0.840	− 0.156	− 0.388	− 0.596	− 0.224	− 0.135	− 0.275	− 0.420	− 0.237	− 1.938
Q3	0.290	0.047	0.035	0.224	0.264	0.117	0.052	0.043	0.125	0.010	− 0.039	− 0.011	− 1.003
*p* value	0.404	0.129	0.234	1.000	1.000	0.294	0.056	0.212	0.664	0.184	**0.048**	0.068	**0.001**
*Bifidobacterium*													
Median	− 0.066	0.531	− 0.068	0.244	− 0.168	− 0.252	− 0.391	0.283	− 0.538	− 0.115	0.441	0.124	− 0.855
Q1	− 0.191	0.231	− 0.116	0.080	− 0.521	− 0.331	− 0.473	0.138	− 0.690	− 0.320	0.171	− 0.096	− 1.003
Q3	0.203	0.580	0.213	0.418	0.224	− 0.186	− 0.248	0.462	− 0.023	0.027	0.598	0.269	− 0.719
*p* value	0.766*	0.002*	0.930*	0.161*	0.255*	0.036*	0.003*	0.008*	0.013*	0.123*	0.005*	0.166*	< 0.001*
*C. coccoides*													
Median	− 0.037	0.641	0.104	− 0.580	− 0.026	− 0.168	− 0.406	− 0.092	− 0.391	− 0.088	0.369	0.000	− 0.327
Q1	− 0.126	0.312	− 0.133	− 0.871	− 0.304	− 0.270	− 0.648	− 0.227	− 0.484	− 0.409	0.220	− 0.112	− 0.766
Q3	0.219	0.951	0.225	− 0.235	0.257	0.050	− 0.114	0.136	− 0.165	0.065	0.635	0.332	− 0.084
*p *value	0.862*	0.002*	0.722*	0.003*	0.608*	0.234	**0.014**	0.356*	0.001*	0.177*	0.009*	0.384*	0.014*

Data are presented in log_10_ of 16S rRNA gene copy number/ml of faecal slurry; 1-sample Wilcoxon test was used for non-parametric data and a paired t-test for parametric data (indicated with asterisk); with bold fonts are displayed statistically significant differences (*p * < 0.05)

*SUCR* sucralose, *MDX* maltodextrin, *STEV* stevia, *CNMD* cinnamaldehyde, *CMC* carboxymethyl cellulose, *P80* polysorbate 80, *CGN* carrageenan, *SB* sodium benzoate, *SS* sodium sulphite, *TIO* titanium dioxide, *ASP* aspartame-based sweetener, *TP* toothpaste, *DET* detergent

## Discussion

It has become increasingly accepted that a diverse gut microbiome with high production of SCFA, particularly butyric acid, is an independent biomarker of host health. It is also known that diet influences the gut microbiome structure and function, including its fibre fermentation capacity [32]. However, relatively little is known about what effect that food additives, artificial sweeteners and accidental exposure to domestic hygiene products might have on the gut microbiome. As our diet has become more industrialised and is expected to become even more so to sustain food availability, it is important to understand the beneficial or detrimental effect food additives may have on the gut microbiome, and by extension to host health, to guide current and future use.

**Fig. 7:**
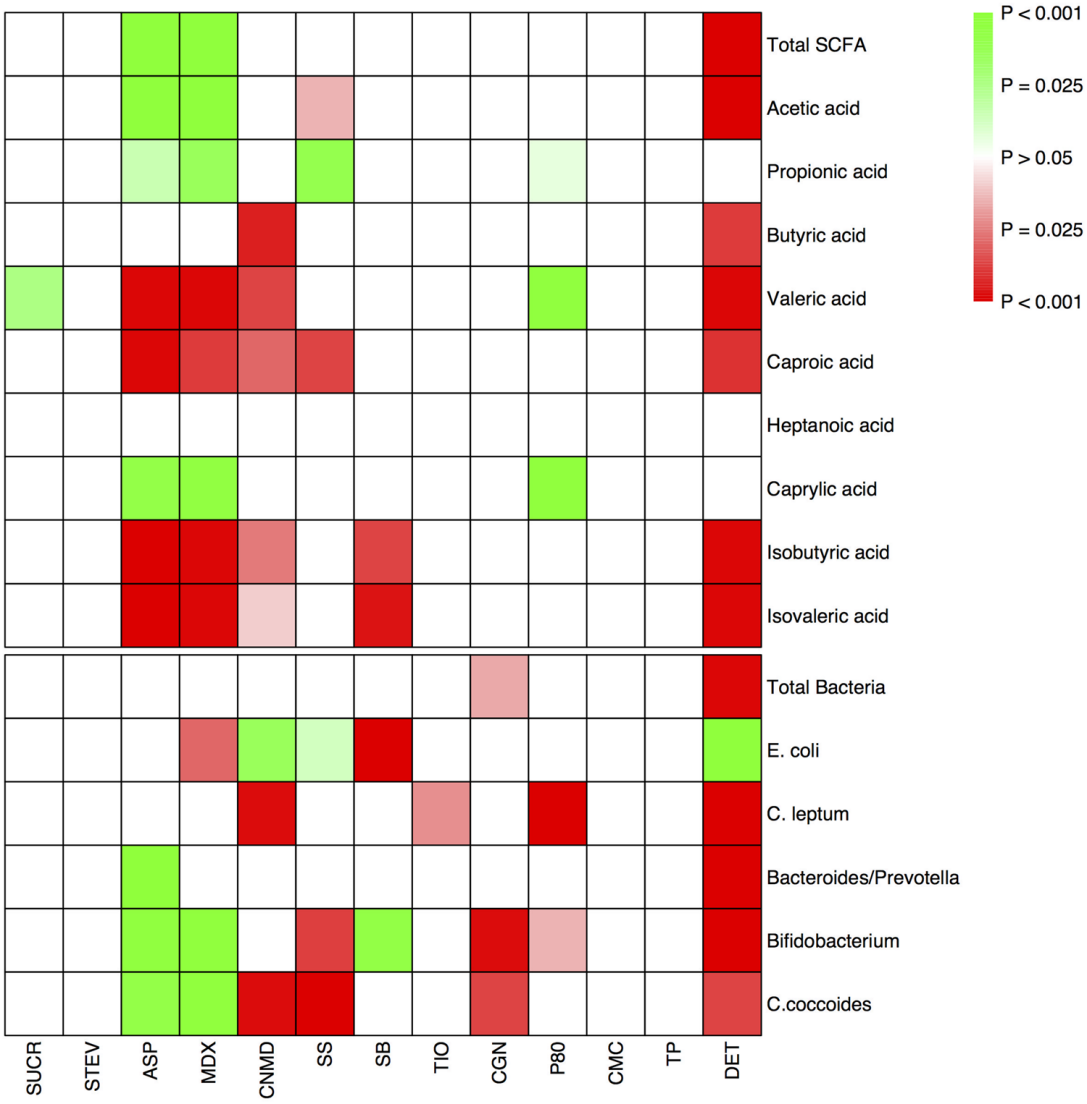
Heatmap illustrating the summary effects of food additives, artificial sweeteners and domestic hygiene products on net production of total and individual short chain fatty acids and concentration of total and major bacterial groups. *0H* baseline, *CTRL* control, *SUCR* sucralose, *STEV* stevia, *ASP* aspartame based sweetener, *MDX* maltodextrin, *CNMD* cinnamaldehyde, *SS* sodium sulphite, *SB* sodium benzoate, *TIO* titanium dioxide, *CGN* carrageenan-kappa, *P80* polysorbate-80, *CMC* carboxymethyl cellulose, *TP* toothpaste, *DET* detergent*. *Red indicates a decrease and green an increase in the concentration of short chain fatty acids or bacterial groups

**Fig. 8:**
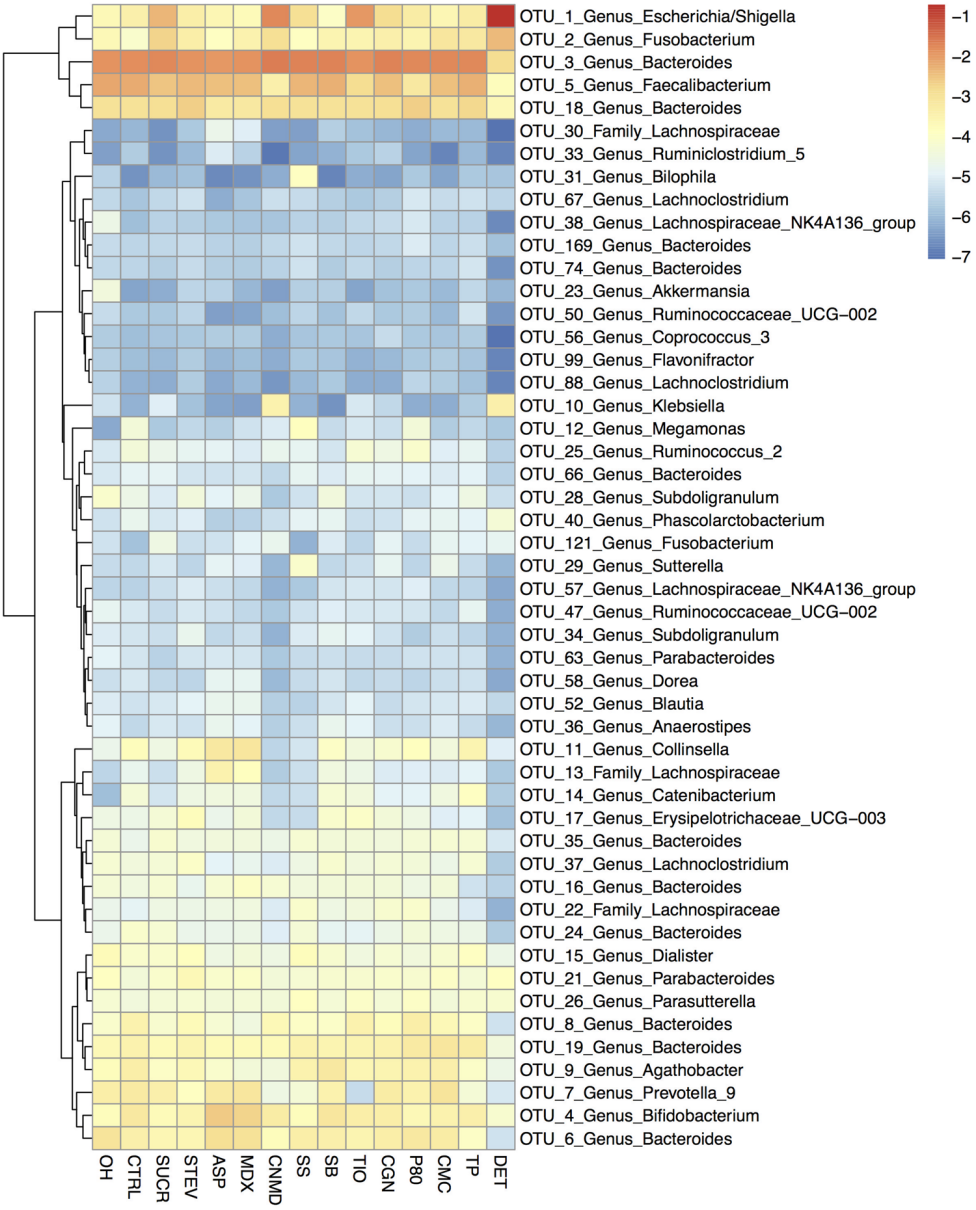
Heatmap illustrating the effects of food additives, artificial sweeteners and domestic hygiene products on mean relative abundance of the top 50 dominant bacterial OTUs across all samples. *0H* baseline, *CTRL* control, *SUCR* sucralose, *STEV* stevia, *ASP* aspartame based sweetener, *MDX* maltodextrin, *CNMD* cinnamaldehyde, *SS* sodium sulphite, *SB* sodium benzoate, *TIO* titanium dioxide, *CGN* carrageenan-kappa, *P80* polysorbate-80, *CMC* carboxymethyl cellulose, *TP* toothpaste, *DET* detergent,* OTU *Operational Taxonomic Unit

This study measured the effect of thirteen commonly used food additives, artificial sweeteners, and domestic hygiene products on the healthy gut microbiome composition and its fermentation capacity using *in-vitro* human microbiome batch fermentations. Changes in the ability of the gut microbiome to ferment fibre and produce SCFA and quantitative changes in major bacterial groups were measured and the summary results of this study are presented in Fig. 7. In addition to these analyses, the global microbiome composition and community structure were characterised using 16S rRNA gene amplicon sequencing as displayed in Fig. 8. Six of the additives affected the production of SCFA, five influenced the global microbiome community structure and nine altered the concentration of dominant microbial groups. Only toothpaste, stevia and carboxymethyl cellulose showed no or minimal effects on the broad composition and fermentation capacity of the faecal microbiome. However, for the additives for which an effect was observed, changes in microbiome composition and SCFA concentrations varied considerably among them; in terms of both the microorganisms or SCFA affected as well as the direction of this effect. Thus, this study highlights that the gut microbiome is modifiable in different ways by different additives. These variable effects of various food additives also suggest that their impact on the gut microbiome needs to be studied separately for each, in combination with each other, and in addition to other macronutrients, micronutrients and fibre in our diet.

There is increasing interest in the effect of the food industrialisation on human health and particularly on non-communicable disease, such as inflammatory bowel disease and diabetes [10, 20]. In previous studies, these effects were associated directly or indirectly with the microbiome of the large bowel. A food additive can affect gut homeostasis by influencing either the gut microbiome, the mucus layer or both. Carrageenan-kappa, upon consumption, has been associated with an increased prevalence of intestinal lesions in animal models [33], highlighting a detrimental effect on the mucosal barrier. Recent evidence from experiments in mice shows that this effect may be mediated by changes in the abundance of *Akkermansia muciniphila*, a potent anti-inflammatory bacterium. The results of the current study show that similar effects were observed with inhibition in the growth of *Bifidobacterium* and *B. coccoides* cluster, members of which have beneficial effects for the host [34]. Similarly, the dietary emulsifiers carboxymethyl cellulose and polysorbate-80 have been proposed to directly alter human microbiome composition and *ex-vivo* gene expression, potentiating intestinal inflammation [21]. Although in our current study no effect of carboxymethyl cellulose was seen on the fermentation capacity or on shifts in major bacterial groups, polysorbate-80 decreased the growth of *Bifidobacterium* and *C. leptum* and the relative abundance of other Firmicutes as confirmed by the results of both qPCR and 16S rRNA gene sequencing. Inorganic sulphite salts are frequently used to stop fermentation in wine and beer as well as antioxidants in food. This bacteriostatic effect of sodium sulphite was observed for members of the genus *Bifidobacterium* and the cluster *B. coccoides* and this effect may give a growth advantage to *E. coli* and *Bilophila wadsworthia* which gain energy through sulphite respiration [35]. Irwin et al. have previously described the bactericidal effects of sodium sulphite on probiotic-type bacteria, common members of the human gut microbiome [36]. The exact opposite effects were observed for the growth of *Bifidobacterium*, the cluster *B. coccoides* and *E. coli* when either maltodextrin or the aspartame-based sweetener was present. This is likely to be because maltodextrin is an artificially produced glucose polymer which, if not absorbed in the small intestine, has prebiotic properties in the colon [37]. Therefore, the increase in the probiotic genus *Bifidobacterium* and B. coccoides and the corresponding decrease in *E. coli* is most likely due to the fact that the former two use maltodextrin for growth [38, 39] instigating fermentation, production of acetic acid and creating an acidic environment in which *E. coli* growth is suppressed. Interestingly, changes in the absolute concentration of these three dominant bacterial groups, quantified with qPCR, were not in parallel the absence of effects observed using next generation sequencing. Sodium benzoate has been shown to decrease plasma ammonium levels by reducing glycine metabolism to treat patients with urea-cycle-disorder and acute hyperammonaemia [40, 41]. Use of sodium benzoate in this study increased the beneficial *Bifidobacterium* but reduced *E. coli* and the concentration of BCFA, suggesting that protein fermentation and potentially production of ammonia from bacterial metabolism in the gut is diminished. Similar to maltodextrin, these effects were not observed with in-depth characterisation of the microbiome using 16S rRNA sequencing. However, discordant results are to be expected as qPCR provides an absolute quantification of broader groups of bacteria and 16S rRNA sequencing offers proportional representation of the overall microbial community. The aspartame-based sweetener we used in this study was rich in maltodextrin in addition to, aspartame and acesulflame potassium. This, therefore, prevented the study of aspartame in isolation. However, the absence of major differences between the maltodextrin and the aspartame-based sweetener suggests that most of the effect seen on the gut microbiome comes from maltodextrin with no major contributions of aspartame and acesulfame potassium; at least in the amount we tested in this experiment which equals 8% of the estimated daily intake which might carry-over to the gut.

Crohn’s disease has been characterised by a gut microbiome with a reduced number of Firmicutes, such as species belonging to C. leptum, Bifidobacterium and Bacteroidetes and an increase in Proteobacteria, particularly *E. coli* strains with adherent and invasive properties [22, 24]. Interestingly, the addition of cinnamaldehyde, a cinnamon ingredient, or dishwashing detergent increased the *E. coli* and decreased the *C. leptum* and *B. coccoides* growth. A similar effect was also observed for polysorbate-80 with a diminished abundance of butyrate-producing species and increase in a species of *Bilophila*, a hydrogen sulphide producer implicated in colitis in IL-10 knockout mice [42]. Assuming that the gut microbial dysbiosis seen in patients with Crohn’s disease is a primary defect of the disease, and such species are implicated in disease pathogenesis, these findings suggest that consumption of cinnamon-containing food, polysorbate-80 and accidental ingestion of residual detergent on crockery and utensils may exacerbate dysbiosis and influence disease outcomes. Dishwashing detergent contains surfactants, which lower the surface tension, potentially causing degradation of mucus layer and the mucosal barrier to break-down thus potentially affecting microbial composition [43] particularly microbes which are adjacent to epithelial cells. Many Firmicutes like *Faecalibacterium* and *Subdoligranulum* are butyric acid-producing bacteria; hence the diminishing production of butyric acid here coincides with the decline in the concentration and abundance of these taxa with the addition of cinnamaldehyde, and dishwashing detergent. Firmicutes constitute a large proportion of the bacteria in the human gut microbiome, therefore, a significant change to the composition and functionality found within this phylum could, in theory, have detrimental consequences to the host. Butyric acid, for example, is the preferable energy substrate for the colonocytes and regulates regulatory T cells which play an important role in cell-mediated immunity [44]. A similar effect on *C. leptum* was seen for polysorbate-80 and a modest one for titanium dioxide. Collectively this evidence proposes that these additives could exacerbate the microbial dysbiosis seen in inflammatory bowel disease.

This study looked at the effect of food additives, artificial sweeteners and domestic hygiene products on the gut microbiome composition and fibre fermentation capacity in healthy human individuals, using batch fermentations with human faecal inoculum; thus, complementing previous research in animals. Although in the current study the SCFA and microbiome composition profile of the control, following 24 h fermentation, are in accordance to those that occur in the human gut, batch fermentation is a snapshot and not an exact simulant of human gut physiology and its complex dynamics [45, 46]. This may explain some of the discrepancies between the findings of this study and previous research [10]. Batch faecal fermentations do, however, provide crucial preclinical data, under well-controlled experimental conditions. They enable exploration of various additives at the same time and the direct effect on the gut microbiome in isolation of the host effect; hence bridging the gap between animal research and human trials. The data generated from this study offer important insights on where future research on additives should be directed, using animal experiments and human randomised controlled trials. In our case, this may be relevant for cinnamaldehyde, polysorbate-80, sodium sulphite, sodium benzoate, sucralose and dishwashing detergent but not for carboxymethyl cellulose, and stevia. While maltodextrin and the aspartame-based sweetener influenced the gut microbiome composition and production of SCFA, they did not induce dysbiosis and their effect might be considered favourable by inhibiting the growth of *E. coli*, thus promoting *Bifidobacterium* and correspondingly increasing the production of acetic acid and propionic acid. This bifidogenic effect of maltodextrin, an artificial glucose polymer has been observed previously too [47]. These findings are in contrast to evidence suggesting that maltodextrin induces dysbiosis promoting gut inflammation [10]. Such discrepancies might be explained by broad differences in the methodology applied among studies and the fact that in the current study we explored the effect of maltodextrin on the gut microbiome in isolation of the host and gut physiology. However, maltodextrin is the main source of carbohydrate in proprietary feeds used for the amelioration of gut inflammation with exclusive enteral nutrition in active Crohn’s disease [48, 49]. This reproducible clinical evidence challenges our current perceptions on the role of maltodextrin on gut inflammation.

This study contributes to the limited knowledge on the effect of food additives, artificial sweeteners and domestic hygiene products on the human gut microbiome composition and fibre fermentation capacity. We have shown that the presence of certain additives changed the microbial composition, and this became similar to the gut microbiome seen in individuals with either inflammatory bowel disease or obesity. For other additives, their effects were counterintuitive and opposite to animal research, implicating them in gut inflammation, and by proxy to human inflammatory bowel disease [10, 50]. This study underpins the importance of evaluating each additive separately and not grouped by their functional class. Here, we lay the groundwork for future research into individual additives on the gut microbiome composition and its fermentation capacity measured over a longer time period both in public health research and in the context of therapeutic interventions in patients with established dysbiosis, including patients with inflammatory bowel disease.

## Electronic supplementary material

Below is the link to the electronic supplementary material.

Supplementary file1 (DOCX 22 kb)

Supplementary file2 (DOCX 16 kb)

Supplementary file3 (DOCX 365 kb)

Supplementary file4 (DOCX 753 kb)
