# Supplementary material for: The impact of food additives, artificial sweeteners and domestic hygiene products on the human gut microbiome and its fibre fermentation capacity
Source: Eur J Nutr. 2019 Dec 18;59(7):3213–30. doi: 10.1007/s00394-019-02161-8 (PMC7501109; doi:10.1007/s00394-019-02161-8)
Supplement: Supplementary file 1 — Supplementary file1 (DOCX 22 kb) [file 394_2019_2161_MOESM1_ESM.docx]

**Online resource 1:** Description and amount of 13 food additives, artificial sweeteners and domestic hygiene products used in the in-vitro batch faecal fermentation studies.

| Functional  class | Additive | Product | Main ingredients | % estimated daily intake/ Acceptable daily intake | Reference |
| --- | --- | --- | --- | --- | --- |
| Sweeteners | Sucralose | BULKPOWDERS TM, SUCRALOSE | Sucralose | 50% | FDA |
|  | Stevia | ViaSweet TM  STEVIA LIQUID | Stevia rebaudiana (95% steviol glycosides) | 50% | EFSA |
|  | Canderel^TM^ | Canderel TM | Maltodextrin, 1.02% aspartame, 0.68% acesulflame-k | 8% | FDA |
| Thickener | Maltodextrin | Bodybuilding WarehouseTM | Maltodextrin | 0.83% | FDA |
| Flavoring agent | Cinnamaldehyde | SIGMA-ALDRICH ^TM^ (C80687) | 99% *trans-*Cinnamaldehyde | 50% | WHO |
| Emulsifiers | Carboxymethyl cellulose | ACROS^TM^ (332631000) | Carboxymethyl cellulose, sodium salt  Mw ~250,000, degree of substitution 1.2 | 27% | JECFA |
|  | Carrageenan | Special-ingredients ^TM^ | Carrageenan kappa | 8.9% | EFSA |
|  | Polysorbate 80 | SIGMA ^TM^ (P1754) | Tween 80 | 27% | JECFA |
| Preservatives | Sodium benzoate | MINERALS-WATER ^TM^, | Sodium benzoate, food grade | 50% | EFSA |
|  | Sodium sulfite | Fluka ^TM^ (S0505) | Sodium sulfite | 50% | EFSA |
| Colorants | Titanium dioxide | Bee Beautiful ^TM^ | Titanium dioxide | 50% | JECFA |
| Hygiene products | Toothpaste | Toothpaste | Proprietary ingredients including Sodium fluoride, Sodium monofluorophosphate,  phenoxyethanol | 100% | (Barnhart et al., 1974) |
|  | Detergent | Dishwashing liquid | Anionic surfactants, non-ionic surfactants, methylisothiazolinone, phenoxyethanol | 100% | (Mercurius-Taylor, Jayaraj and Clark, 1984) |

Barnhart WE, Hiller LK, Leonard GJ, Michaels SE. Dentifrice usage and ingestion among four age groups. J Dent Res. 1974;53(6):1317-22

Mercurius-Taylor LA, Jayaraj AP, Clark CG. Is chronic detergent ingestion harmful to the gut? Br J Ind Med. 1984;41(2):279-81.
