# Supplementary material for: The impact of food additives, artificial sweeteners and domestic hygiene products on the human gut microbiome and its fibre fermentation capacity
Source: Eur J Nutr. 2019 Dec 18;59(7):3213–30. doi: 10.1007/s00394-019-02161-8 (PMC7501109; doi:10.1007/s00394-019-02161-8)
Supplement: Supplementary file 2 — Supplementary file2 (DOCX 16 kb) [file 394_2019_2161_MOESM2_ESM.docx]

**Online resource 2**: Primers and probes used in quantitative PCR of major bacterial groups

| **Target** |  | **Sequence 5’ -3’** |
| --- | --- | --- |
| Total Bacteria | probe  forward primer  reverse primer | CTT GTA CAC ACC GCC CGT C  CGG TGA ATA CGT TCC CGG  TAC GGC TAC CTT GTT ACG ACT T |
| Bacteroides | probe  forward primer  reverse primer | AAG GTC CCC CAC ATT G  CCT TCG ATG GAT AGG GGT T  CAC GCT ACT TGG CTG GTT CAG |
| Bifidobacterium | probe  forward primer  reverse primer | CTC CTG GAA ACG GGT G  CGG GTG AGT AAT GCG TGA CC  TGA TAG GAC GCG ACC CCA |
| C. coccoides group | probe  forward primer  reverse primer | CGG TAC CTG ACT AAG AAG  GAC GCC GCG TGA AGG A  AGC CCC AGC CTT TCA CAT C |
| C leptum group | probe  forward primer  reverse primer | CAC AAT AAG TAA TCC ACC  CCT TCC GTG CCG CAG TTA  GAA TTA AAC CAC ATA CTC CAC TGC TT |
| E coli | probe  forward primer  reverse primer | TAT TAA CTT TAC TCC CTT CCT CCC CGC TGA A  CAT GCC GCG TGT ATG AAGA A  CGG GTA ACG TCA ATG AGC AAA |

Gerasimidis K, Bertz M, Hanske L, et al. Decline in presumptively protective gut bacterial species and metabolites are paradoxically associated with disease improvement in pediatric Crohn's disease during enteral nutrition. Inflammatory bowel diseases 2014;20(5):861-71
