# Supplementary material for: The impact of food additives, artificial sweeteners and domestic hygiene products on the human gut microbiome and its fibre fermentation capacity
Source: Eur J Nutr. 2019 Dec 18;59(7):3213–30. doi: 10.1007/s00394-019-02161-8 (PMC7501109; doi:10.1007/s00394-019-02161-8)
Supplement: Supplementary file 3 — Supplementary file3 (DOCX 365 kb) [file 394_2019_2161_MOESM3_ESM.docx]

**Online resource:** Proportional ratio of individual short chain fatty acids at baseline and following 24h batch faecal fermentation of fibre with food additives, artificial sweeteners and domestic hygiene products.


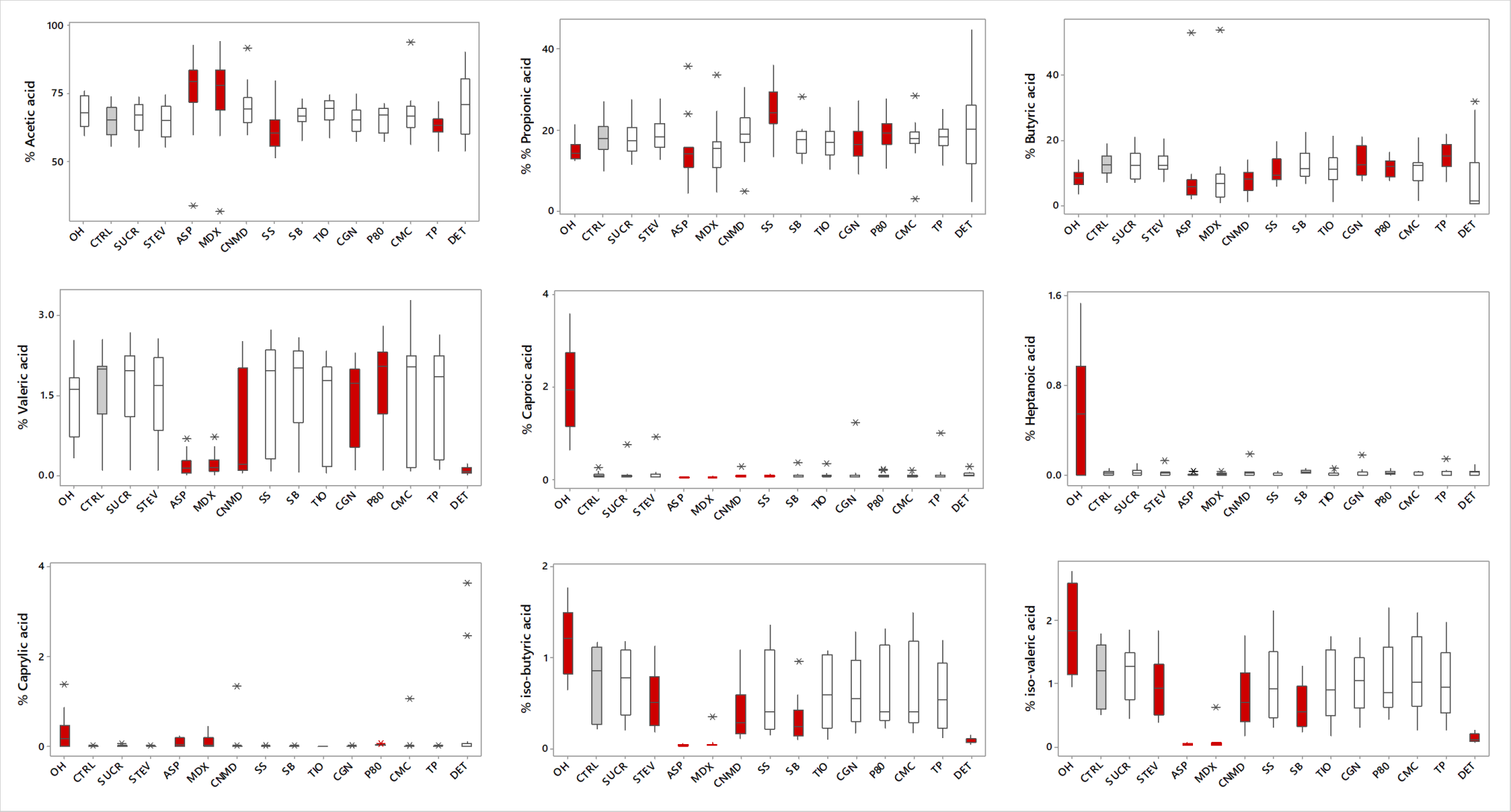


*Red filling boxplot indicates significant difference (p<0.05) compared with the CTRL (displayed with grey filling boxplot); 0H: baseline, CTRL: control, SUCR: sucralose, STEV: stevia, ASP: aspartame based sweetener, MDX: maltodextrin, CNMD: cinnamaldehyde, SS: sodium sulphite, SB: sodium benzoate, TIO: titanium dioxide, CGN: carrageenan-kappa, P80: polysorbate-80, CMC: carboxymethyl cellulose, TP: toothpaste, DET: detergent*
