# Supplementary material for: The impact of food additives, artificial sweeteners and domestic hygiene products on the human gut microbiome and its fibre fermentation capacity
Source: Eur J Nutr. 2019 Dec 18;59(7):3213–30. doi: 10.1007/s00394-019-02161-8 (PMC7501109; doi:10.1007/s00394-019-02161-8)
Supplement: Supplementary file 4 — Supplementary file4 (DOCX 753 kb) [file 394_2019_2161_MOESM4_ESM.docx]

**Online resource 3**:

Fig: The effect of food additives, artificial sweeteners and domestic hygiene products on bacterial family relative abundance

Fig: The effect of food additives, artificial sweeteners and domestic hygiene products on genus relative abundance
